# Interobserver reproducibility of fully quantitative pixel-wise analysis of clinical CMR perfusion imaging

**DOI:** 10.1186/1532-429X-16-S1-P350

**Published:** 2014-01-16

**Authors:** Hannah Conn, Li-Yueh Hsu, Susanne Winkler, Allison D Ta, Kim-Lien Nguyen, Peter Kellman, Sujata M Shanbhag, Marcus Y Chen, W Patricia Bandettini, Andrew E Arai

**Affiliations:** 1 National Heart, Lung, and Blood Institute, National Institutes of Health, Bethesda, Maryland, USA

## Background

Quantitative first pass cardiac magnetic resonance (CMR) perfusion imaging has shown excellent interobserver agreement at a sector level in healthy volunteers and patients. In this study, we compare the myocardial blood flow (MBF) estimates in sector-wise and pixel-wise analysis. We also study the interobserver variability in pixel-wise MBF estimates from patients with coronary artery disease (CAD).

## Methods

First pass CMR imaging was performed on 29 patients with known or suspected CAD (15 females, age 54.9 ± 14.3 years). Twenty of the patients, defined as the normal group, had minimal or no stenosis ( < 30% by computed tomographic angiogram) and nine patients, defined as the CAD group, had significant CAD ( > 70% stenosis by invasive coronary angiography). All patients were scanned on a 1.5T scanner using a steady state free precession imaging sequence for regadenoson stress perfusion followed by rest perfusion 20 minutes later. Two observers independently traced the myocardial regions of interest in the mid-ventricular slice and quantified the MBF in sector-wise and pixel-wise analyses by a model-constrained deconvolution approach. Pixel-wise MBF estimates were averaged to six transmural sectors to compare with sector-wise analysis. Pearson correlation, Bland-Altman analysis, and paired student t-test were used to compare the results.

## Results

**Figure 1:**
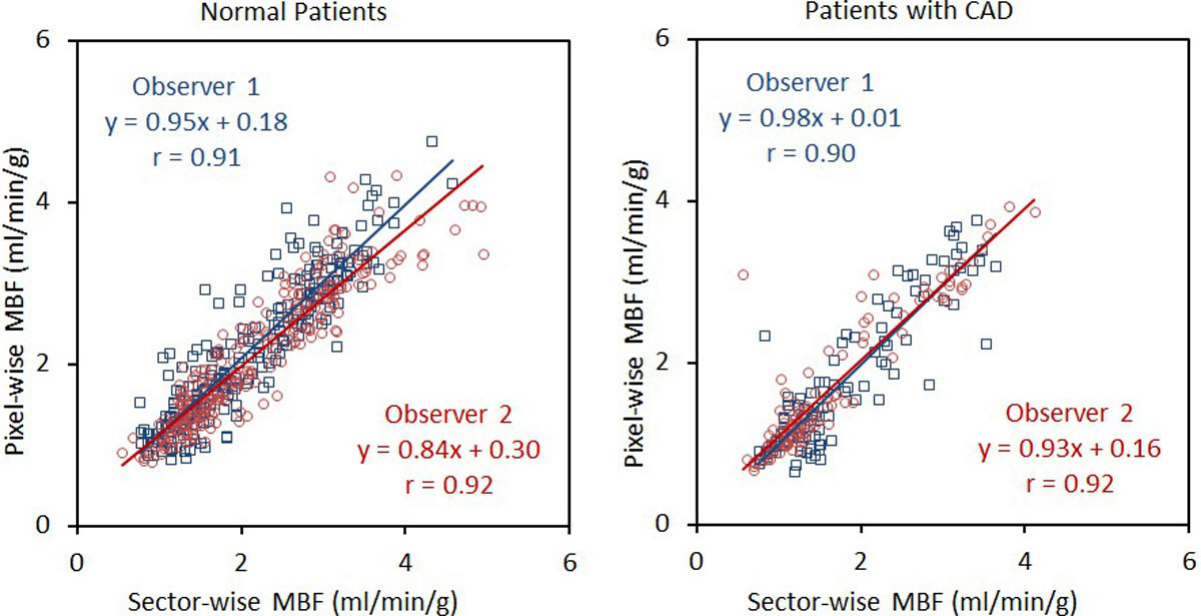
**Excellent correlation between pixel-wise vs. sector-wise MBF quantification**.

**Figure 2:**
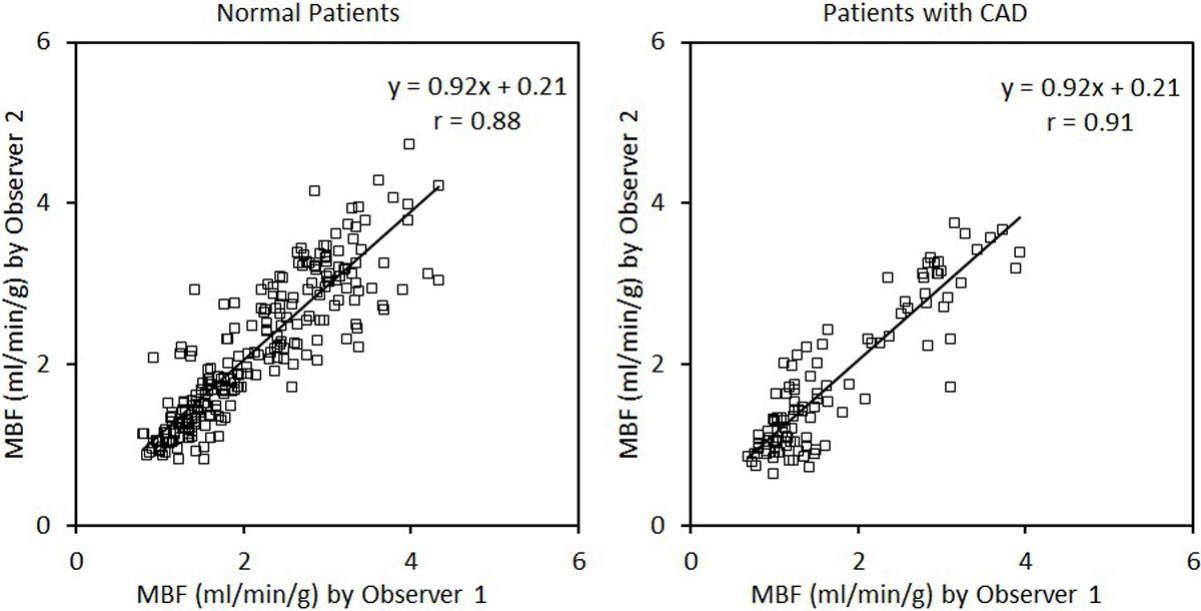
**Excellent agreement of pixel-wise MBF quantification between two independent observers**.

There was excellent correlation between pixel-wise vs. sector-wise MBF quantification for the 20 normal and nine CAD patients (Figure 1). In both patient groups, Bland-Altman analysis showed no significant bias between the two methods of quantification (mean bias from 0.01 to 0.12 ml/min/g). Limits of agreement were good (2SD range from 0.26 to 0.98 ml/min/g, p = NS for all comparison). Interobserver agreement of pixel-wise MBF was excellent for both normal and CAD groups (Figure 2). The interobserver agreement was good (2SD range from 0.38 to 0.96 ml/min/g, p = NS for all comparisons) with no significant interobserver bias (mean bias from 0.02 to 0.15 ml/min/g). The stress MBF in the ischemic zone of patients with > 70% stenosis was 1.72 ± 0.75 ml/min/g by pixel-wise analysis which was significantly lower than remote MBF (2.85 ± 0.74 ml/min/g, p < 0.001).

## Conclusions

Clinical first pass CMR perfusion can be quantified at the pixel level and the results agree well with sector-wise comparison. There is an excellent interobserver agreement in pixel-wise quantification of patients with CAD.

## Funding

This research was supported by the Intramural Research Program of the National Heart, Lung, and Blood Institute, National Institutes of Health.

